# Descriptive epidemiology of gastrointestinal non-Hodgkin's lymphoma in a population-based registry

**DOI:** 10.1038/sj.bjc.6690307

**Published:** 1999-04

**Authors:** K A Gurney, R A Cartwright, E A Gilman

**Affiliations:** Leukaemia Research Fund Centre for Clinical Epidemiology, University of Leeds, 17 Springfield Mount, Leeds, LS2 9NG, UK

**Keywords:** epidemiology, non-Hodgkin's lymphoma, gastrointestinal, time-trend analysis

## Abstract

The incidence of non-Hodgkin's lymphoma (NHL), particularly at certain extranodal sites, has been demonstrated to be rising, at least in the USA, more than for any other malignancy. One of the major sites of extranodal NHL is the gastrointestinal tract, though little is known of its epidemiological characteristics. Over an 8-year period (1986 to 1993) 1069 primary gastrointestinal NHL cases were reported to the Leukaemia Research Fund Data Collection Survey which covers many parts of England and Wales. Age-standardized incidence rates of gastrointestinal NHL at all sites (0.58/10^5^ per year), gastric (0.24/10^5^ per year), small bowel (0.17/10^5^ per year) and large bowel (0.06/10^5^ per year) confirmed that the UK has the lowest rates of gastrointestinal NHL in Europe. An excess of males was observed at all ages and for all sites. Time-trend analyses showed annual increases in incidence rates for gastric (6.3%) and small bowel (5.9%) NHL although a concomitant decrease in gastrointestinal NHL of unknown site suggested that at least part of these increases had resulted from more accurate diagnoses. Overall, the incidence of gastrointestinal NHL significantly increased by 2.7% per annum and was limited to the population aged over 50 years in this series. © 1999 Cancer Research Campaign


British Journal of Cancer (1999) **79**(11/12), 1929–1934
© 1999 Cancer Research Campaign
Article no. bjoc.1998.0307

### Descriptive epidemiology of gastrointestinal non-Hodgkin's lymphoma in a population-based registry

**KA Gurney, RA Cartwright and EA Gilman**

Leukaemia Research Fund Centre for Clinical Epidemiology, University of Leeds, 17 Springfield Mount, Leeds LS2 9NG, UK

**Summary** The incidence of non-Hodgkin's lymphoma (NHL), particularly at certain extranodal sites, has been demonstrated to be rising, at least in the USA, more than for any other malignancy. One of the major sites of extranodal NHL is the gastrointestinal tract, though little is known of its epidemiological characteristics. Over an 8-year period (1986 to 1993) 1069 primary gastrointestinal NHL cases were reported to the Leukaemia Research Fund Data Collection Survey which covers many parts of England and Wales. Age-standardized incidence rates of gastrointestinal NHL at all sites ($0.58/10^5$ per year), gastric ($0.24/10^5$ per year), small bowel ($0.17/10^5$ per year) and large bowel ($0.06/10^5$ per year) confirmed that the UK has the lowest rates of gastrointestinal NHL in Europe. An excess of males was observed at all ages and for all sites. Time-trend analyses showed annual increases in incidence rates for gastric (6.3%) and small bowel (5.9%) NHL although a concomitant decrease in gastrointestinal NHL of unknown site suggested that at least part of these increases had resulted from more accurate diagnoses. Overall, the incidence of gastrointestinal NHL significantly increased by 2.7% per annum and was limited to the population aged over 50 years in this series.

**Keywords:** epidemiology; non-Hodgkin's lymphoma; gastrointestinal; time-trend analysis

Most non-Hodgkin's lymphoma (NHL) originates in the lymph nodes or other lymphoid tissues. However, a significant proportion of NHL has a primary extranodal site such as the gastrointestinal tract, skin or central nervous system. Primary gastrointestinal NHL is the commonest extranodal localization, representing around one-third of all extranodal cases in most series (Zucca et al, 1997). Recently it has been shown that incidence rates for NHL have been increasing for many years with this trend being more prevalent in extranodal disease than in nodal NHL (Devesa and Fears, 1992; Zheng et al, 1992). It is, as yet, uncertain as to whether this is a real increase or due to more efficient case registration and improved diagnoses although it has been suggested that the increase in incidence rates for gastric lymphoma in the United States is independent of coding and diagnostic procedures (Severson and Davis, 1990).

In 1984 the Leukaemia Research Fund began the Data Collection Survey (DCS), a specialist population-based register covering neoplastic haematological diseases in parts of England and Wales based on diagnoses from collaborating haematologists and histopathologists. Data collected by this Survey have been shown to be more accurate, as they reflect modern clinical and haematopathological opinions, and more complete than comparative National Health Service cancer registry data (Alexander et al, 1989; Cartwright et al, 1997).

This paper describes the epidemiology of gastrointestinal NHL and investigates the changes in incidence in gastric and other intestinal NHL in those areas of England and Wales covered by the DCS from 1986 to 1993.

*Received 14 August 1998*
*Revised 16 October 1998*
*Accepted 27 October 1998*

*Correspondence to:* RA Cartwright

### MATERIALS AND METHODS

The DCS is a specialist registry of lymphomas, leukaemias and related disorders held by the Leukaemia Research Centre for Clinical Epidemiology in Leeds. This survey has been collecting data since 1 January 1984, with the aim of providing high quality data for population-based epidemiological studies (Cartwright et al, 1990). The geographical area of case collection covers approximately half of England and Wales. Newly diagnosed cases are reported directly to Leeds from collaborating histopathologists and haematologists and registrations completed by trained data collection clerks. In addition, regular checks are made with NHS cancer registries for the areas concerned and any missed cases registered after review.

NHL cases were registered by the DCS only after histopathological review by a minimum of two histopathologists with a specialist interest in lymphomas. Data from the DCS and the cancer registries for the years 1989–93 were compared using log-linear models and case coverage for all NHL was estimated to be nearly 96% (Cartwright et al, 1997).

Gastrointestinal NHL was defined as cases where the initial symptoms and predominant disease were confined to the gastrointestinal tract (Lewin et al, 1978) including the pancreas and oesophagus. Cases with a gastrointestinal site discovered during staging or other clinical investigation of NHL and mesenteric localizations were excluded from the study. In accordance with DCS criteria, cases were registered as primary gastrointestinal NHL, after histopathological review with no evidence of nodal involvement.

The localization of gastrointestinal NHL was further classified into gastric, small bowel and large bowel NHL, and gastrointestinal (GI) tract, site unknown, NHL by review of the histopathology reports. The gastric site comprised those cases where the disease was localized in the stomach or in the stomach and intestine. Small

**Table 1** Age-standardized incidence rates and site distribution for gastrointestinal non-Hodgkin's lymphoma, 1986–1993

| Site | Male | | | Female | | | Total | | | M:F ratio |
|---|---|---|---|---|---|---|---|---|---|---|
| | Rate[a] | Number of cases | % | Rate[a] | Number of cases | % | Rate[a] | Number of cases | % | |
| Gastric | 0.29 | 252 | 41.1 | 0.20 | 211 | 46.2 | 0.24 | 463 | 43.3 | 1.5 |
| Small bowel | 0.22 | 167 | 27.3 | 0.13 | 126 | 27.6 | 0.17 | 293 | 27.4 | 1.7 |
| Ileum | | 29 | | | 23 | | | 52 | | |
| Duodenum | | 11 | | | 7 | | | 18 | | |
| NOS | | 127 | | | 96 | | | 223 | | |
| Large bowel | 0.09 | 71 | 11.6 | 0.04 | 48 | 10.5 | 0.06 | 119 | 11.1 | 2.3 |
| Rectum | | 25 | | | 18 | | | 43 | | |
| Colon | | 21 | | | 15 | | | 36 | | |
| Caecum | | 20 | | | 9 | | | 29 | | |
| NOS | | 5 | | | 6 | | | 11 | | |
| GI tract, site unknown | 0.13 | 107 | 19.9 | 0.06 | 65 | 15.7 | 0.10 | 172 | 16.1 | 2.2 |
| Gastrointestinal NOS | | 84 | | | 60 | | | 144 | | |
| Intestine NOS | | 23 | | | 5 | | | 28 | | |
| All sites[b] | 0.75 | 612 | 100.0 | 0.43 | 457 | 100.0 | 0.58 | 1069 | 100.0 | 1.7 |

[a]Rate directly standardized to the World Standard population (cases/100 000/year). [b]Includes gastrointestinal NHL with the primary site localized to the pancreas (*n* = 14) and oesophagus (*n* = 8).

**Table 2** Classification of gastrointestinal NHL by grade and age

| Age range (years) | Sex | Low-grade | High-grade | T-cell | All graded cases | Unclassified cases | Total |
|---|---|---|---|---|---|---|---|
| 0–14 | M | 0 | 11 (100.0%)[a] | 0 | 11 | 1 (8.3%)[b] | 12 |
| | F | 0 | 1 (100.0%) | 0 | 1 | 0 | 1 |
| 15–29 | M | 2 (10%) | 16 (80.0%) | 2 (10%) | 20 | 1 (4.8%) | 21 |
| | F | 0 | 1 (50.0%) | 0 | 1 | 1 (50.0%) | 2 |
| 30–49 | M | 26 (36.6%) | 40 (56.3%) | 5 (7.1%) | 71 | 9 (11.3%) | 80 |
| | F | 14 (35.0%) | 24 (60.0%) | 2 (5.0%) | 40 | 10 (20.0%) | 50 |
| 50–69 | M | 81 (37.0%) | 117 (53.4%) | 21 (9.6%) | 219 | 47 (17.7%) | 266 |
| | F | 72 (45.3%) | 76 (47.8%) | 11 (6.9%) | 159 | 34 (17.6%) | 193 |
| 70+ | M | 76 (40.9%) | 102 (54.8%) | 8 (4.3%) | 186 | 47 (20.2%) | 233 |
| | F | 53 (33.5%) | 89 (56.3%) | 16 (10.1%) | 158 | 53 (25.1%) | 211 |

[a]% of all graded cases. [b]% of total cases.

bowel included duodenum, jejunum, ileum, ileo caecal junction localizations and small intestine not otherwise specified. The large bowel included appendix, caecum, colon, rectum localizations and large intestine not otherwise specified. A separate category of GI tract, site unknown, NHL included cases where the primary site had not been recorded and cases where the site was reported as intestinal or gastrointestinal with no further details.

Gastrointestinal NHL was classified according to the updated Kiel classification and graded as either low- or high-grade malignancy (Stansfeld et al, 1988).

This study was based on all gastrointestinal NHL cases diagnosed between 1 January 1986 and 31 December 1993 and reported to the DCS. Age-specific incidence rates were calculated for males, females and both sexes combined for all gastrointestinal NHL, and individually for gastric, small bowel, large bowel sites and GI tract, site unknown. To calculate age-specific incidence rates, cases were grouped into 5-year age groups with the exception of the final group which was 90 years and older. To facilitate geographical comparisons, rates were standardized by the direct method using the World Standard Population (Parkin et al, 1992) which required adjustment of the final age group to 85 years and older. Incidence trends were evaluated by Poisson regression analysis over the full age range for all gastrointestinal NHL and for the individual sites. Incidence trends were also investigated, where sufficient data were available, using four age ranges (0–29, 30–49, 50–69 and 70+ years) to examine in more detail whether changes in incidence rates were more prominent in some age groups than in others.

### RESULTS

### Incidence of gastrointestinal NHL

A total of 1069 cases of gastrointestinal NHL were diagnosed during the years from January 1986 to December 1993. During this time, 11 334 cases of NHL were diagnosed within the population covered by the Data Collection Survey, thus the gastrointestinal NHL cases represented 9.43% of all NHL cases.

 *© Cancer Research Campaign 1999*

**Table 3** Histological classification of gastrointestinal NHL for individual sites

| Histology | Gastric | Small bowel | Large bowel | GI tract, site unknown |
|---|---|---|---|---|
| Low grade | 153 (43.3%) | 75 (30.4%) | 35 (33.0%) | 54 (33.8%) |
| Lymphoplasmacytoid | 10 | 4 | 3 | 4 |
| Centrocytic | 18 | 12 | 13 | 8 |
| Centroblastic-centrocytic, follicular | 28 | 19 | 5 | 8 |
| Centroblastic-centrocytic, follicular and diffuse | 6 | 9 | 1 | 6 |
| Centroblastic-centrocytic, diffuse | 31 | 14 | 5 | 16 |
| Low grade, unclassified | 60 | 17 | 8 | 12 |
| High grade | 191 (54.1%) | 131 (53.0%) | 62 (58.5%) | 81 (50.6%) |
| Centroblastic | 75 | 38 | 21 | 26 |
| Lymphoblastic, Burkitt's type | 0 | 3 | 3 | 3 |
| Lymphoblastic, convoluted cell | 1 | 0 | 0 | 0 |
| Lymphoblastic, other/unclassified | 6 | 5 | 2 | 7 |
| Immunoblastic | 11 | 12 | 11 | 10 |
| Histiocytic lymphoma | 10 | 11 | 6 | 4 |
| High grade, unclassified | 88 | 62 | 19 | 31 |
| T-cell lymphoma NOS | 9 (2.5%) | 41 (16.6%) | 9 (8.5%) | 6 (3.8%) |
| All classified cases | 353 (100%) | 247 (100%) | 106 (100%) | 141 (100%) |
| Malignant lymphoma, unspecified grade (% of total cases) | 110 (23.8%) | 46 (15.7%) | 13 (10.9%) | 31 (18.0%) |
| Total cases | 463 | 293 | 119 | 172 |

Age-standardized (World standard population) incidence rates for all gastrointestinal NHL and by individual site are presented in Table 1. The overall age-standardized incidence rates for gastrointestinal NHL all sites combined were 0.75 per 100 000 and 0.43 per 100 000 for males and females respectively. Ages ranged between 5 years 1 month and 95 years 9 months (mean = 64 years 10 months, median = 67 years 6 months). An excess in male incidence was found for all gastrointestinal NHL and for the individual sites ranging from a male:female ratio of 1.5:1 for gastric NHL to 2.3:1 for large bowel NHL. This excess in male cases was even more pronounced in children and young adults where 11 of 12 cases in the 0–14 age range and 21 of 23 cases in the 15–29 age range were male (Table 2).

### Site of gastrointestinal NHL

Table 1 also shows that the most common site of gastrointestinal NHL for all age groups was gastric (43.3%) followed by small bowel (27.4%), large bowel (11.1%) with a further 172 cases classified as GI tract, site unknown, NHL (16.1%). However, for children and young adults (< 30 years) small bowel NHL was predominant (44.4%), then GI tract, site unknown (25.0%), gastric (16.7%) and large bowel (13.9%).

### Histology of gastrointestinal NHL

Overall, most gastrointestinal NHL cases in this series were high-grade (44.5%), with 30.4% low-grade, 19.0% unclassified and 6.1% T-cell lymphomas. The distribution of histological subtypes among gastrointestinal sites is given in Table 3. Gastric NHL had a higher proportion of low-grade cases whereas intestinal NHL

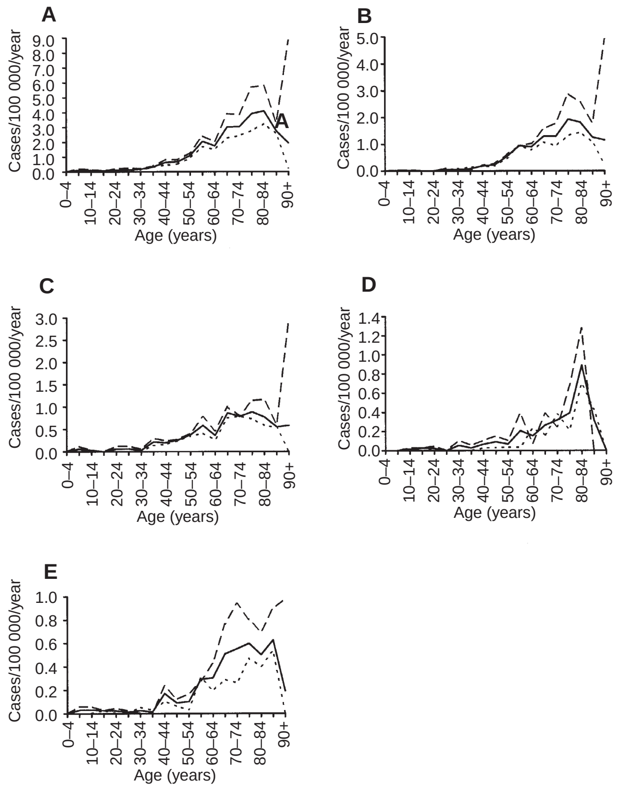

**Figure 1** Age-specific incidence rates, male (– – –), female (- - -) and combined (——), for all gastrointestinal NHL (**A**), gastric NHL (**B**), small bowel NHL (**C**), large bowel NHL (**D**) and GI tract, site unknown, NHL (**E**) in parts of England and Wales, 1986–1993

**Table 4** Time trend analysis of incidence rates of gastrointestinal NHL in parts of England and Wales, 1986–1993

| | Annual change in incidence | | 95% confidence intervals |
|---|---|---|---|
| All gastrointestinal NHL | | | |
| Males | + 2.19% | NS | |
| Females | + 3.16% | NS | |
| Total | + 2.66% | $P$ = 0.047 | 0.03, 5.30 |
| Gastric | | | |
| Males | + 6.88% | $P$ = 0.014 | 1.34, 12.72 |
| Females | + 5.37% | NS | |
| Total | + 6.25% | $P$ = 0.003 | 2.16, 10.51 |
| Small bowel | | | |
| Males | + 5.65% | NS | |
| Females | + 6.08% | NS | |
| Total | + 5.87% | $P$ = 0.023 | 0.78, 11.23 |
| Large bowel | | | |
| Males | – 0.03% | NS | |
| Females | + 3.45% | NS | |
| Total | + 1.44% | NS | |
| GI tract, site unknown | | | |
| Males | – 13.29% | $P$ = 0.001 | – 5.31, – 20.61 |
| Females | – 11.68% | $P$ = 0.026 | – 1.21, – 21.04 |
| Total | – 12.62% | $P$ < 0.001 | – 6.36, – 18.47 |

(small and large bowel cases combined) tended to be high-grade. Over 75% of T-cell lymphomas were found in intestinal NHL cases. For all gastrointestinal NHL the most common histological subtype was centroblastic, accounting for nearly 20% of all classified lymphomas. All childhood, and the majority of young adult, gastrointestinal NHL cases were high-grade malignancies. In children (0–14 years) most cases were lymphoblastic, Burkitt's type, but in the young adults (15–29 years) centroblastic was again predominant. In adults (≥ 30 years old) the distribution of high-grade, low-grade and T-cell gastrointestinal NHL were broadly similar for each age range.

Age-specific incidence curves by sex for all gastrointestinal NHL and by individual site are shown in Figure 1. Incidence rates for both sexes show broadly similar patterns for all sites of gastrointestinal NHL. Rates are very low for the under 30s then rapidly increase to a major peak around 80 ± 5 years of age, except in large bowel NHL where this peak occurs over a narrower age range and GI tract, site unknown, NHL, where the age range is rather broader. All sites appear to show a minor peak in age-specific incidence rates for the 55–60 year age group with the exception of GI tract, site unknown, NHL where the peak occurs much earlier at 40–45 years of age.

### Change in incidence

From 1986 to 1993, incidence of gastrointestinal NHL for both sexes combined increased by 2.66% per annum ($P$ = 0.047; 95% CI, 0.03, 5.30) (Table 4). Over this time period the incidence of NHL classified as having a primary site in the stomach or small bowel showed significant annual increases of 6.25% and 5.87%, respectively whilst that of large bowel NHL remained stable. A highly significant annual decrease of 12.62% in the incidence of GI tract, site unknown, NHL was also observed. Similar values for annual increases and decreases were seen in males and females separately but for the female population these trends were not significant except for GI tract, site unknown, NHL. In males, gastric and GI tract, site unknown, NHL incidence rates showed a significant increase and decrease respectively.

More detailed analyses of the increase in incidence rates from 1986 to 1993 for all gastrointestinal NHL revealed that significant increases were restricted to the older age groups (0–29 years, + 5.5%, NS; 30–49 years, – 2.6%, NS; 50–69 years, + 3.5%, $P$ = 0.09; 70+ years, + 5.3%, $P$ = 0.01; males and females combined). This pattern was also seen for gastric NHL (70+ years, + 8.5%, $P$ = 0.02). Analyses for other sites and age ranges were unreliable due to small numbers of cases.

Time trend analyses for low- and high-grade gastrointestinal NHL at all sites showed no significant changes for either of these groups (low-grade disease showed an annual decrease of 1.7%, NS; high-grade disease showed an annual increase of 1.5%, NS; males and females combined). However, cases with unspecified grade showed an annual increase of 7.15%, $P$ = 0.02 reflecting the relatively high proportion of gastric and small bowel cases with unknown grade.

### DISCUSSION

The majority of studies investigating the epidemiology of gastrointestinal NHL have been based on studies using selective data with the exception of four north European population-based studies [Otter et al, 1989*a* (96 cases); D'Amore et al, 1994 (306 cases); Halme et al, 1997 (61 cases); Ducreux et al, 1998 (78 cases)]. To our knowledge, this study comprising 1069 cases represents the largest population-based series of gastrointestinal NHL. This, along with uniform and highly complete collection of data, are the main strengths of the current study. The principal disadvantages are those shared by similar studies in that it is not known whether all extranodal disease has been included, the site may be vague in some cases and it is not always possible to distinguish a nodal condition infiltrating into the bowel from primary bowel disease.

*© Cancer Research Campaign 1999*

The incidence rates found in this study for gastrointestinal NHL in parts of England and Wales are lower than those reported by previous population-based studies in France, central Finland and Denmark (D'Amore et al, 1994; Halme et al, 1997; Ducreux et al, 1998). This difference is more marked for gastric NHL, where incidence is less than half that found in other European countries, than for intestinal NHL where the rates are more similar to those reported. Incidence rates calculated from cancer registry data for 14 countries by Newton et al (1997) also showed that England and Wales had the lowest rate of gastric NHL of all developed countries and that small intestine NHL rates showed much less variation. These authors calculated incidence rates of 0.21, 0.16 and 0.08 per 100 000 for gastric, small intestine and colon NHL, respectively, which are very similar to the rates found in the present study.

Other epidemiological characteristics of gastrointestinal NHL in the present study were comparable to earlier studies, with nearly half of gastrointestinal NHL cases localized to the stomach, and small bowel and large bowel lymphomas representing just over one quarter and one-tenth of cases, respectively (Halme et al, 1997; Zucca et al, 1997). Gastrointestinal NHL in children and young adults (< 30 years old) was more commonly found in the small bowel rather than the stomach. A hospital-based series reviewed by Weingrad et al (1982) found that 73% of patients had a primary gastric lymphoma but this series did not include children who typically have relatively more intestinal disease than adults. In other studies, a greater proportion of large bowel to small bowel NHL probably reflects the inclusion of mesenteric presentations in the former group, whilst in this study these have been recorded as nodal disease and have not been included in the analyses for gastrointestinal NHL (Otter et al, 1989*b*; Ducreux et al, 1998). In addition, there is considerable evidence that geographical variation exists. Among demographically similar communities in the UK and Italy, large differences in the incidence of gastric lymphoma have been reported (Doglioni et al, 1992). The higher incidence of gastric lymphoma found in Italy was associated with excesses in gastric adenocarcinoma and *Helicobacter pylori* infection which may be involved in the development of both neoplasms. *H. pylori* infection has been implicated in the development of low-grade gastric lymphoma derived from mucosa-associated lymphoid tissue (MALT) (Wotherspoon et al, 1991). In Middle Eastern and Mediterranean populations there is a relatively high incidence of small bowel NHL linked to immunoproliferative alpha-chain disease. A similar hypothesis, involving a putative microorganism, has been postulated to operate in immunoproliferative small intestine lymphomas (Isaacson and Spencer, 1993). The importance of *H. pylori* infection in the aetiology of gastric lymphoma is still uncertain. There have been several reports of successful treatment of low-grade gastric lymphoma with antibiotics alone but MALT formation leading to B-cell clonality and lymphoma may not be linked exclusively to *H. pylori* infection (Sorrentino et al, 1996).

Gastrointestinal NHL is principally a disease of middle age and older with nearly 85% of cases in this series aged over 50 years. Male predominance, found to be around 2:1 depending on the site, has been observed in all series whether population- or hospital-based, although there is as yet no explanation for this excess. The distribution of low- and high-grade gastrointestinal NHLs in this series is similar to that found in previous studies with more cases of high-grade disease than low-grade disease overall. However, there are differences between the individual sites with low-grade NHL more common in gastric disease and high-grade NHL more common in intestinal disease. T-cell lymphomas were found mostly in intestinal NHL cases as expected.

There have been several reports recently that the incidence of NHL malignancies is increasing rapidly, primarily in the developed countries, by between 3–5% per annum (Devesa and Fears, 1992; McNally et al, 1997; Morgan et al, 1997) and that this increase is seen more in extranodal disease than in nodal NHL (Devesa and Fears, 1992; Zheng et al, 1992).

In the period from 1986 to 1993 the incidence of gastrointestinal NHL of all sites in England and Wales increased by more than 2.5% annually. This is the first report of an increase in gastrointestinal NHL in Europe. More detailed analyses showed that this increase was limited to the older age groups with a moderate increase in the 50 to 69-year-old age group and the largest increase in the over 70-year-old group as has been found in the United States for gastric lymphomas (Severson and Davis, 1990). Incidence rates of gastric and small bowel NHL each showed annual increases of around 6%. In the United States an annual increase of 3.7% in small intestinal NHL was found during the period 1974 to 1988 and rises in incidence rates for gastric lymphoma have been reported (Severson and Davis, 1990; Devesa and Fears, 1992).

Epidemiological studies have identified a number of factors which may be of importance in the aetiology of NHL, though not specifically to gastrointestinal NHL. These include exposure to ultraviolet light, to pesticides, solvents and other chemicals and the excess of protein and fat in the diet (Franceschi et al, 1989; Cartwright et al, 1994). Increasing exposure or susceptibility to these environmental risk factors could be responsible for the increasing trend in gastrointestinal NHL. Greater awareness of *H. pylori* may result in more cases of gastrointestinal NHL, and in particular MALT lymphomas, being identified. This series included no data on MALT lymphomas, however, as these lymphomas are typically low-grade malignancies and no increase in low-grade disease was seen over the period from 1986–1993 it seems unlikely that this could account for the observed increase in gastric NHL incidence. Alternatively, endoscopic biopsies and diagnostic techniques may have led to increased accuracy in the diagnosis of gastrointestinal NHL. Worldwide, incidence rates of gastric cancer have declined over recent years (Coleman et al, 1993). This has been cited as evidence against an improvement in diagnoses but neoplasms previously diagnosed as gastric carcinomas may now be more accurately diagnosed as lymphomas.

The most obvious conclusion in this series would be that improved diagnosis of gastrointestinal NHL in the 1990s may be responsible, at least in part, for the observed increases in gastric and small bowel NHL, as a concomitant decrease in the incidence of gastrointestinal NHL of unknown primary site was observed. However, a real increase in gastric and small bowel lymphoma cannot be excluded given that an annual increase of more than 2.5% in the incidence of gastrointestinal NHL at all sites was observed in England and Wales between 1986 and 1993.

### ACKNOWLEDGEMENTS

The Leukaemia Research Fund has financed all aspects of the work for this publication. Numerous haematologists, histopathologists, radiotherapists and consultants in other specialities are thanked for their continued efforts to facilitate case ascertainment; these are acknowledged in detail elsewhere (Cartwright et al, 1990). We gratefully acknowledge the invaluable assistance and

© Cancer Research Campaign 1999

high standard of case recording achieved by the Leukaemia Research Fund data clerks.

### REFERENCES

Alexander FE, Ricketts TJ, McKinney PA and Cartwright RA (1989) Cancer registration of leukaemia and lymphoma: results of a comparison with a specialist register. *Comm Med* **11**: 81–89

Cartwright RA, Alexander FE, McKinney PA and Ricketts TJ (1990) *Leukaemia and Lymphoma. An Atlas of Distribution within Areas of England and Wales 1984–1988*. Leukaemia Research Fund: London

Cartwright RA, McNally R and Staines A (1994) The increasing incidence of non-Hodgkin's lymphoma (NHL): the possible role of sunlight. *Leuk Lymphoma* **14**: 387–394

Cartwright RA, McNally RJQ, Rowland DJ and Thomas J (1997) *Descriptive Epidemiology of Leukaemia and Related Conditions in Parts of the United Kingdom 1984–1993*. Leukaemia Research Fund: London

Coleman MP, Estève J, Domiecki P, Arslan A and Renard H (1993) *Trends in Cancer Incidence and Mortality*. International Agency for Research on Cancer: Lyon

D'Amore F, Brincker H, Grønbæk K, Thorling K, Pedersen M, Jensen MK, Andersen E, Pedersen NT and Mortensen LS (1994) Non-Hodgkin's lymphoma of the gastrointestinal tract: a population-based analysis of incidence, geographic distribution, clinicopathological features, and prognosis. *J Clin Oncol* **12**: 1673–1684

Devesa SS and Fears T (1992) Non-Hodgkin's lymphoma time trends: United States and international data. *Cancer Res* **52**(Suppl): 5432s–5440s

Doglioni C, Wotherspoon AC, Mochine A, de Boni M and Isaacson PG (1992) High incidence of primary gastric lymphoma in northeastern Italy. *Lancet* **339**: 834–835

Ducreux M, Boutron M-C, Piard F, Carli P-M and Faivre J (1998) A 15-year series of gastrointestinal non-Hodgkin's lymphomas: a population-based study. *Br J Cancer* **77**: 511–514

Franceschi S, Serraino D, Carbone A, Talamini R and La Vecchia C (1989) Dietary factors and non-Hodgkin's lymphoma: a case-control study in the northeastern part of Italy. *Nutr Cancer* **12**: 333–341

Halme L, Mecklin J-P, Juhola M, Krees R and Palmu A (1997) Primary gastrointestinal non-Hodgkin's lymphoma. *Acta Oncol* **36**: 69–74

Isaacson PG and Spencer J (1993) Malignant lymphoma and autoimmune disease. *Histopathol* **22**: 509–510

Lewin KJ, Ranchod M and Dorfman RF (1978) Lymphomas of the gastrointestinal tract. *Cancer* **42**: 693–707

McNally RJQ, Alexander FE, Staines A and Cartwright RA (1997) A comparison of three methods of analysis for age-period-cohort models with application to incidence data on non-Hodgkin's lymphoma. *Int J Epidemiol* **26**: 32–46

Morgan G, Vornanen M, Puitinen J, Naukkarinen A, Brincker H, Olsen J, Coeburgh JW, Vrints LW, Clayden D, McNally R, Jacj A, Carli PM, Petrella T, Tomino R, D'Lollo S, Barchielli A and Cartwright R (1997) Changing trends in the incidence of non-Hodgkin's lymphoma in Europe. *Ann Oncol* **8** (Suppl 2): 549–554

Newton R, Ferlay J, Beral V and Devesa SS (1997) The epidemiology of non-Hodgkin's lymphoma: comparison of nodal and extranodal sites. *Int J Cancer* **72**: 923–930

Otter R, Bieger R, Kluin M, Hermans J and Willemze R (1989*a*) Primary gastrointestinal non-Hodgkin's lymphoma in a population-based registry. *Br J Cancer* **60**: 745–750

Otter R, Gerrits WBJ, VD Sandt MM, Hermans J and Willemze R (1989*b*) Primary extranodal and nodal non-Hodgkin's lymphoma. *Eur J Cancer* **25**: 1203–1210

Parkin DM, Muir CS, Whelan SL, Gao YT, Ferlay J and Powell J (1992) *Cancer Incidence in Five Continents. Vol VI*. International Agency for Research on Cancer: Lyon

Severson RK and Davis S (1990) Increasing incidence of primary gastric lymphoma. *Cancer* **66**: 1283–1287

Sorrentino D, Ferraccioli GF, DeVita S, Avellini C, Beltrami CA, Labombarda A, Bernardis V, De Biase F, Trevisi A, Pivetta B, Boiocchi M and Bartoli E (1996) B-cell clonality and infection with *Helicobacter pylori*: implications for development of gastric lymphoma. *Gut* **38**: 837–840

Stansfeld AG, Diebold J, Kapanci Y, Kelényi, Lennert K, Mioduszewska O, Noel H, Rilke F, Sundstrom, Van Unnik JAM and Wright DH (1988) Updated Kiel classification for lymphomas. *Lancet* **i**: 292–293

Weingrad DN, Decosse JJ, Sherlock P, Straus D, Lieberman PH and Filippa DA (1982) Primary gastrointestinal lymphoma. *Cancer* **49**: 1258–1265

Wotherspoon AC, Ortiz-Hidalgo C, Falzon MR and Isaacson PG (1991) *Helicobacter pylori*-associated gastritis and primary B-cell gastric lymphoma. *Lancet* **338 i**: 1175–1176

Zheng T, Mayne ST, Boyle P, Holford TR, Liu WL and Flannery J (1992) Epidemiology of non-Hodgkin lymphoma in Connecticut. *Cancer* **70**: 840–849

Zucca E, Roggero E, Bertoni F and Cavalli F (1997) Primary extranodal non-Hodgkin's lymphomas. Part 1: Gastrointestinal, cutaneous and genitourinary lymphomas. *Ann Oncol* **8**: 727–737

 *© Cancer Research Campaign 1999*
